# Refusal of Retreatment With Topical 5-Fluorouracil Among Patients With Actinic Keratosis: Qualitative Analysis

**DOI:** 10.2196/39988

**Published:** 2023-02-15

**Authors:** Rohan Singh, Sarah McCain, Steven R Feldman

**Affiliations:** 1 Department of Dermatology Wake Forest School of Medicine Winston Salem, NC United States; 2 Wake Forest School of Medicine Winston Salem, NC United States

**Keywords:** actinic keratosis, solar keratosis, topical 5-fluorouracil, fluorouracil, topical treatment, cancer, carcinoma, neoplasm, oncology, pharmacotherapy, pharmacological, malignant, malignancy, tolerability, adherence, preference, opinion, attrition, attitude, perception, qualitative, skin, dermatology, lesion

## Abstract

**Background:**

Actinic keratosis (AK) is a common premalignant skin lesion, and topical 5-fluorouracil (5-FU) is commonly used in field-directed therapy. However, 5-FU is associated with frequent local skin reactions.

**Objective:**

This study aimed to qualitatively assess experiences among patients with AK who refuse retreatment with 5-FU.

**Methods:**

Semistructured interviews were conducted with 10 adult participants who had received treatment with 5-FU for AK between January 1, 2017, and January 1, 2020, and refused future treatment with 5-FU. Results were analyzed using qualitative research methods.

**Results:**

Although most participants had low concern upon having received a diagnosis of AK, most felt that treatment is very important. When initiating treatment with 5-FU, most cited recommendation by their health care professionals as the primary motivator and initially had low concern regarding treatment. The side effects associated with treatment were physically and psychosocially burdensome for most participants and led to temporary lifestyle adjustments. After treatment, most did not believe that their health care provider prepared them for treatment or were unsure. While half of the participants felt that 5-FU helped treat AKs, half were either unsure, due to premature discontinuation, or did not think that 5-FU treated their AKs.

**Conclusions:**

5-FU is one of the most commonly prescribed treatments for AKs, yet most patients experienced both a physical and psychosocial burden with the treatment. Inability to assess efficacy due to premature discontinuation secondary to 5-FU–related reactions is common, and shared decision-making, navigating treatment options, and taking into account patient preferences may be critical to help assure better adherence and outcomes. Although our study was limited by input from participants who refused future treatment with 5-FU, most stated that they would still continue to seek treatment for AKs in the future and would consider other topical treatments, especially if associated with a milder tolerability profile.

## Introduction

Actinic keratosis (AK) is a common premalignant lesion clinically presenting as a scaly, red papule on sun-exposed areas of the skin [1]. AKs are treated using a variety of different treatment modalities to prevent progression to invasive squamous cell carcinoma [2]. Treatments can either be lesion-directed (for limited lesions) or field-directed therapies that target areas of skin with multiple AKs and clinical evidence of field cancerization [2].

Topical 5-fluorouracil (5-FU) is a commonly used field-directed therapy to treat AK [2]. It blocks DNA synthesis by inhibiting thymidylate synthetase, which targets the rapidly growing dysplastic cells involved in AK pathogenesis and causes the cells to undergo apoptosis [3]. Topical 5-FU may also be the most efficacious field-directed treatment for AK according to a network meta-analysis [4]. While topical 5-FU is commonly used to treat AKs, many patients experience adverse effects, especially burdensome local skin reactions (LSRs), including inflammatory erythema, scaling, crusting, swelling, erosion, and localized pain [3,5]. LSRs are seen in up to 90% of patients [5]. These can be bothersome and can interfere with activities of daily living and social engagements, leading to a negative impact on quality of life [6].

Adverse effects, frequent dosing, and prolonged duration of treatment can lead to poor adherence, early discontinuation of treatment, and may deter patients from future retreatment with 5-FU [5]. Few studies have assessed adherence to topical 5-FU, and LSRs may discourage patients from seeking treatment for future AKs; some patients refuse to use 5-FU a second time, potentially increasing the risk of malignant transformation [5,7]. Moreover, there is limited literature assessing patient experience with 5-FU treatment. Previous studies using interventions, such as educational videos, to improve patient satisfaction have also not been successful [8]. To further characterize the adverse effects of topical 5-FU and its impact on future use, we qualitatively assessed patient experience among those who stated they would refuse future treatment with 5-FU.

## Methods

### Recruitment

Participants who received a clinical diagnosis of AK (ICD10: L57) and had received treatment with 5-FU between January 1, 2017, and January 1, 2020, were identified through a retrospective review of patients from the Atrium Health Wake Forest Baptist Dermatology Clinic. Potential participants were contacted for assessment, and telephone interviews were conducted with 10 participants who refused future treatment with 5-FU. Multiple reports analyzing patient cohort sizes in qualitative research have suggested the utility of a small sample size, particularly among homogenous populations, such as ours, to address the research question. Specifically, certain commentators have suggested a sample size of 10 to be adequate to sample among a homogenous population [9,10]. Participant recruitment was additionally terminated once thematic saturation was achieved. Each participant received a unique identification code as “Pt#.”

### Interviews

Data were collected through audio-recorded semistructured interviews. Interviews lasted between 7 and 33 minutes. Participants were asked about details regarding 5-FU use (indication, dose, duration, outcome, and condition response), medication regimen details (additional medications taken), impact that treatment has had on patient life, and adverse effects experienced. Every participant was asked 22 core questions (Textbox 1). Additional questions were asked to clarify or expand upon responses.

**Interview questionnaire listing the questions asked during semistructured interviews with the study participants.**

1. Can you please tell me how you felt when you were diagnosed with actinic keratosis (AK)?
2. How important to you is receiving the proper treatment?
3. Why do you consider treatment important/not important?
4. Please, tell me about your previous experiences with treatment—if you had any?
5. When receiving treatment, did you get any support? From whom? Did it help?
6. Have you previously (in the past) discontinued your treatment for AK? Why?
7. Please, tell me about your experience of using the topical 5-fluorouracil (5-FU) cream?
8. What made you use the medications?
9. How did you feel about the 5-FU treatment?
10. Can you tell me about the challenges you experienced?
11. Did you have any concerns about the treatment? Can you describe them for me?
12. Did the use of 5-FU help treat your AKs?
13. How did the 5-FU affect your everyday life? In particular, has this affected social activities or your job responsibilities?
14. How did the 5-FU treatment make you feel about your appearance?
15. What effects did taking the medicine have on you?
16. Can you describe your side-effects? (When did they occur? How long did they last? Which one was the worst? Did anything make the side effects better?)
17. How did you feel during that time?
18. Were you prepared by your health care provider to manage the side effects? Can you describe how that affected you?
19. Did the side effects cause you to stop the treatment?
20. Did the side effects outweigh the benefits of treating your AK?
21. In the future, would you be willing to try other topical treatments for AK? Why/why not?
22. In the future, would you seek treatment for AK? Why/why not?

### Method of Analysis

The semistructured participant interviews were recorded, carefully reviewed, and then transcribed verbatim upon conclusion of participant interviews. Results were then analyzed using both descriptive statistics and a qualitative research method. The interview transcriptions were initially reviewed by study team members (RS, SM, and SRF) and then coded by 2 members of the study team (RS and SM). Preliminary codes were initially identified using open coding and collaborative analysis. The codes were then clustered in accordance with categories, and themes were then used for further data refinement per conventional qualitative analysis as described in literature [11]. Representative quotations were extracted for illustrative purposes. NVivo (version 11; QSR International) software was used to analyze data and aid in data management.

### Ethical Considerations

This study received ethics approval from the Wake Forest University of Health Science’s institutional review board (IRB00077121).

## Results

### Overview

A total of 1276 potential participants were identified (mean age 68 years, 69% male, and all of them were White). All of the 10 participants included in this study identified as male and were White (mean age 66 years, range 39-89 years). Participant interviews revealed 4 major themes centered on how participants felt and their concerns about a diagnosis of AK, motivation to start 5-FU treatment, experience during treatment and perception of preparation, and how treatment affected perception of AK and future care.

### Participants’ Concerns and Input When Diagnosed With AK

There was low concern among most participants upon having received a diagnosis of AK, although some were shocked or unhappy with their diagnosis. While most were not overly concerned regarding their diagnosis, almost all participants considered treatment very important, and most specifically cited the underlying carcinogenic etiology of the lesion as the reason for the criticality of treatment. Other less commonly stated reasons for concern included general health concerns and embarrassment associated lesion appearance (Table 1).

**Table 1:** Representative quotes exemplifying participant reactions to their diagnosis of actinic keratosis and their initial motivation to use topical 5-fluorouracil cream.

Theme	Quote
Concern and input when diagnosed with actinic keratosis	*Well, I don't want to have this disease or any other if I can help it. And of course they want to remove it if I had it….I think I was shocked to hear the word cancer associated with my health.* [Pt #2] *I’ve had them for years and I treated them in the past. And so it was just something that from time to time I will treat them to so they don't get any worse.* [Pt #5] *I have known people to die of skin cancer at an alarming rate. If you were to ask me what I know most of them to die from, cancer would be probably in the top five.* [Pt #6]
Motivation to start topical 5-fluorouracil treatment	*No, I didn't have any concerns. I mean you're talking to someone who about always trusted my doctors to give me medication that was going to be what I needed and that it would work and never imagined that there would be you know a shortfall in quality of what I got.* [Pt #2] *It was just the doctor's recommendation initially.* [Pt #3] *No, not really. I trusted the physician that was prescribing and I read did some research on myself.* [Pt #7]

### Patient Motivation to Start 5-FU Treatment

When participants were asked why they initiated therapy with 5-FU, almost all stated the recommendation by their health care provider (HCP) as the reason. Moreover, almost all participants stated they had no or very low concern at the time 5-FU was recommended and prescribed by their HCP. Trust in the prescribing HCP was a pervasive subtheme that participants mentioned to explain the lack of concern. Most participants had received prior interventions for their AKs, and only 3 participants stated they had never received any previous treatment for AK. Among the treatments referred, cryosurgery and other surgical interventions were the most common. Photodynamic therapy and treatment with another topical medication were also referenced. Only one participant stated having previously discontinued treatment for AK (Table 1).

### Experience During Treatment and Perception of Preparation

Almost all participants experienced difficulty during treatment with 5-FU, specifically due to symptoms from 5-FU–related adverse effects. Pain or burning at the site of the application was most commonly reported by participants. Other common adverse effects that caused patient difficulty during treatment included red or inflamed skin, peeling, sloughing, and flaking of the skin, and blistering of the skin at the site of application. Most participants stated that symptom onset occurred within days of 5-FU application and resolved within days to 2 weeks after application cessation. However, there was a particular subset of participants who reported residual effects of the medication for months after application cessation (Table 2).

Treatment with 5-FU also was associated with a negative impact on participant self-perception. Particularly, many participants stated they felt self-conscious or embarrassed while receiving treatment, particularly in those who experienced visible inflammation in their skin with associated blistering or peeling of the skin at the site of application. The treatment, and associated visible symptoms, also caused a pervasive impairment in the social activities of participants, although most stated that the treatment itself did not physically limit them from completing daily activities. A subset of the participants also experienced impaired sleep during treatment (Table 2).

When asked regarding challenges faced while on treatment, most participants referenced 5-FU–associated pain and an overall lifestyle adjustment they had to make while on therapy. Specifically, participants stated issues with application of the medication, especially around bedtime to avoid smearing of contents, and preparation for their social appearance (Table 2).

Overall, all participants felt they were not adequately prepared by their HCP on what to expect while on treatment and few were also unsure whether their HCP had adequately informed them before treatment. During treatment, participants reported that they either received support from their family and their physician or their staff, from just their family, or just from their physician or staff. Some participants, however, stated that they received no support while on treatment (Table 2).

**Table 2:** Representative patient quotes regarding treatment experience and preparation.

Theme	Quote
Experience during treatment	*It made me look very sick. Cause my face and arms were extremely red. And at times had blisters on them. *[Pt #1] *It was difficult because it was painful and unsightly….if I were to say bad, that would mean to me very, very painful.* [Pt #2] *[Which side effect that you described earlier was the worst sir?] Probably burning, tingling sensation on the scalp. Much similar to say, a sunburn feeling.* [Pt #8] *You get that fluorouracil cream, you get diagnosed with getting whatever that treats … and, um, it was terrible… So I mean, I'm happy for treatment, but it's hard, some hard treatment.*[Pt #9]
Symptom timeline	*I'd say at least five, four to five days. That's when I noticed it starting to turn red, it stinged, it peeled. At the end of the treatment, my last day that I used it just seemed like stayed red for a long time. And I would I would say approximately a good month before it ever started diminishing and then vanished probably within that next month, just real slow.* [Pt #5] *Probably within 12 to 24 hours at first application. [And how long did they last?] They probably stopped about 3-4 days after my last application.* [Pt #8] *It starts itching and burning. So those were some side effects. High levels of pain once you get into the, you know, it started hurting probably past the itching and burning phase, you know day five to seven, and at night, you know at night I get home and you know I think it was I was reapplied on it twice a day. I can't remember but I remember it hurts when you go to bed. So you have sometimes have trouble sleeping, because of the pain and you don't want to move too much. You know put your face on a pillow like the last thing you want to do.* [Pt #9]
Daily activities and psychosocial impact	*It made me look very sick. Cause my face and arms were extremely red. And at times had blisters on them.* [Pt #1] *I think it did dramatically. Yeah, when you can't sleep at night that's pretty serious. I think I was retired at the time. So it didn't affect my job responsibilities, but it’s certainly affecting my everyday life because I was just miserable.* [Pt #3] *I didn’t let it affect me in any way, I went on and did everything that I needed to do. You know, there was there were times it probably caused me to not get decent sleep, but other than that, you know, it was painful. Nobody knew about it. I didn't tell anybody or let anybody know or let it inhibit anything I needed to do. And I have a high tolerance for pain, which is a whole other story.* [Pt #4] *Well it didn't make me feel good. If my skin is blistered, and I can’t go out with friends and everything and then obviously it doesn't make me feel real good.* [Pt #7] *It was hard when I used it. Yeah, I mean, just like anything else, you look like a zombie... People probably stare at me, you know? But it's, it's I guess it's all how you take it. You know, I've had so many hard things that it doesn't, you know, I just kept doing... I mean, socially, I probably went out less and did things less just, I don't want to have to have that conversation.* [Pt #9]
Challenges while on treatment	*It starting to turn red, it stinged, it peeled. At the end of the treatment, my last day that I used it just seemed like stayed red for a long time. And I would I would say approximately a good month before it ever started diminishing and then vanished probably within that next month, just real slow.* [Pt #5] *It's just that it's a commitment on my part whenever I started it, that I would not be venturing out in public too much because it does look like a leper or something. I mean, it's like more like leprosy.* [Pt #7] *Well there was a period of time where I would have very little exposure outside. Generally, it said it was recommended that I should do it in the winter. It set strict adherence to not going out in sunlight, to touch my skin. Applying it twice a day and made sure I wore gloves when I put it on so as not to get it, you know rubbing my eyes for example or any mucosal membrane. And then also using pillow cases such that I didn’t have to be concerned about the pillowcases becoming discolored or damaged that type of thing. When I applied it before bedtime.* [Pt #8] *Only one thing that's good about it is it's easy to put on. But after you put it on it starts... I mean you can't touch it. You know after a few days your face is so tender, and I only put on my face that’s why I keep talking about my face, but so tender you definitely ain’t going in the sunshine. It's like a Marvel movie the way it just burns when you get in the sun. It's crazy. And then once your face, my face started swelling up to the point where I was having, I couldn't chew or having trouble just talking, couldn't smile. A smile on my face was gonna crack open. Those are some of the basic challenges, I guess. And itching and burning, of course, I mean, the whole time.* [Pt #9]
Level of preparedness	*Well, I think the entire staff, the dermatology department were very supportive, you know, not just the doctors but the nurses.* [Pt #2] *No, I didn't get any support. It was like the job that I had was to survive it.* [Pt #4] *No, they didn't mention anything like that. It didn't bother me with my doctor. I mean, it just didn't work for me. I don't know. I'll tell him when I go back.* [Pt #10]

### How 5-FU Treatment Affected Patient Perception of AK and Future Care

Participants stated having mixed feelings while receiving treatment with 5-FU. While some stated treatment was fine or a minor convenience, other participant responses included feeling self-conscious. Including those who stated that treatment was a minor inconvenience, more participants felt uncomfortable and self-conscious during treatment with 5-FU. Particularly, of the participants who felt uncomfortable and self-conscious, some stated that treatment was very difficult for them (Table 3).

**Table 3:** Representative quotes regarding patient perception of treatment success and satisfaction.

Theme	Quote
Convenience of treatment	*I do remember they were saying a lot of it was pre-cancer. And that's what this particular drug was supposed to do, so you know, deal with. So it was it was sort of, I had to do something that was difficult. And so how I felt about it.* [Pt #2] *Well, generally I felt great, because I didn't you know, this was is like putting a Band-Aid on and you got some underneath the Band-Aid that hurts real bad. And you do everything you got to do you just keep doing it. You know it didn't inhibit anything that I did at all. Because I just got going I wouldn’t let it slow down the things I had to do.* [Pt #4] *Like crap. That would be the mental side, the physical side, not so bad.* [Pt #7] *It wasn't... let’s put it this way it was a minor annoyance. And at times you, by focusing on other things in life I didn't even really pay much attention.* [Pt #8]
Perception of treatment effectiveness	*Short answer, no. The full answer is somewhat partially in some areas, like my neck is significantly improved over what it was. And there's very small areas there. Down my chest on either side of my sternum, on the left side, there's very little, but there's a rim that that runs probably a four inch line with little very small spots of it left. On the right side there are larger spots over a wider area, and the area below my breasts at the bottom of my sternum on the right hand side there's a fairly significant area that's kind of appeared afterwards.* [Pt #4] *Yes, it definitely works. It's just a question of whether that is the best solution.* [Pt #7] *It only provided very, very short-term results and would need to have to be repeated frequently.* [Pt #8] *It's 50/50. It's as much as you can take, because it ends up...you're going to have to do something. So either I took as much as I could and I said you know whatever's next I have to take next because I couldn't finish.* [Pt #9]
Patient satisfaction with treatment	*In the long run, of course, I'm happy that the doctor seems to believe that it helped, and I trust doctors, you know.* [Pt #2] *Well, the positive effects were that it treated the cancer. I mean, for me it's hard though because I wore glasses. On my face and anywhere my glasses touched, I had to wear like a like a napkin, on my nose bridge. And I mean, putting it on every day and wash my face and there's a constant burning that goes with it that kind of affects your behavior just by your attitude. You kind of like get tired of that burning. And then it starts swelling and you can't move your face from the people staring at you and everything else. It's a, I mean it’s a process. You know? It's a difficult process to go through.* [Pt #9]
Likelihood to seek future treatment	*Well, of course, I'd be willing to this I was told that the side effects were much more minimal. Then the ones in the fluorouracil. I’m not interested in going through that again.* [Pt #4] *I think if a dermatologist recommended another way of treating it, I would definitely look into other ways, yes.* [Pt #5] *I would consider it depending on the ease of use, as well as a discussion about effectiveness.* [Pt #8] *I said yes, I would be willing to try after consulting with my doctor. But if it’s going to do the same thing the other cream done, I'm not interested.* [Pt #10]

There were mixed responses from participants about whether treatment with 5-FU helped treat AK. Half of the participants believed that 5-FU helped treat their AKs, while the other half of the participants were not sure or stated that it did not help. Inability to assess treatment responses due to premature discontinuation of 5-FU was a particular subtheme. Specifically, 4 participants stated that side effects from 5-FU use caused them to stop treatment (Table 3).

Although some participants were satisfied with treating AK owing to its potentially carcinogenic nature, mental health impairment and lifestyle inconvenience were other particular subthemes. There was a pervasive belief among participants that it was either difficult to assess or the benefits of treatment were not worth the side effects associated with 5-FU (Table 3).

Despite all participants stating that they would refuse future treatment with 5-FU for AK, most stated that their experience with 5-FU would not deter them for seeking future alternative treatment, and they would be open to other topical treatments, specifically if associated with fewer side effects (Table 3).

## Discussion

### Principal Findings

All patients in our cohort refused future retreatment with 5-FU. Despite an overall low concern when diagnosed with AK, most participants believed that treatment was important. There are multiple treatment options for AKs, including both surgical and field-directed treatments, and cryosurgery and 5-FU are the most commonly prescribed therapies [2,12]. While cryosurgery is intended to treat isolated or a few lesions, topical treatments, such as 5-FU, are more efficacious to treat both clinical and subclinical AKs [2,12,13]. However, 5-FU is associated with frequent LSRs, and patients with a greater number of AKs at baseline may be predisposed to severe LSRs [14,15].

Most participants stated that they had had low concern with therapy before initiating treatment and trusted the recommendation of their HCP. However, after completing treatment, most participants stated that they were either unsure or were not adequately prepared by their HCP. Moreover, there were mixed responses regarding the efficacy of 5-FU treatment. Specifically, many participants did not believe that treatment helped their AKs. Inability to assess effectiveness due to premature discontinuation secondary to 5-FU–related adverse events (AEs) was also a common theme. There was a pervasive theme that the side effects associated with treatment were not worth the benefits.

Altogether, participants experienced both physical and psychosocial burden secondary to topical 5-FU treatment. During treatment, 5-FU–associated pain or burning, erythema, peeling, sloughing, and flaking caused difficulty to participants. Particularly, 5-FU–associated reactions had a negative impact on participant self-perception and caused feelings of self-consciousness and embarrassment. Social impairment during treatment, secondary to 5-FU–associated LSRs, was another common theme. Although treatment did not prevent participants’ abilities to complete daily activities, it led to lifestyle changes and limitations. Sleep impairment and prevention of 5-FU smearing onto bedsheets was a notable subtheme.

### Limitations

Our study was limited by input from participants who refused future retreatment with 5-FU, potential recall bias, and lack of an objective measure of treatment burden. Still, most stated that they would still continue to seek alternative treatment for AKs in the future and would still be open to other topical treatments, especially if associated with a better safety and tolerability profile than 5-FU. Nonadherence to treatment is a major cause of treatment failure. Particularly, up to 50% of dermatology patients may be nonadherent to treatment regimens [16]. Ineffective communication about treatment-related AEs may predispose patients to poor adherence [16,17]. Prescription of a topical steroid as needed may also help decrease the burden of intolerable skin irritation secondary to 5-FU [18]. Shared decision-making with patients after discussing the benefits and risks of medicines, in addition to therapeutic options, and appropriate counseling may increase adherence and improve patient outcomes [19,20].

### Conclusions

Most participants believed that AK is important to treat and had low concern regarding 5-FU at baseline. During treatment, participants experienced both physical and psychosocial burden secondary to topical 5-FU treatment. Although our study was limited by input from participants who refused future treatment with 5-FU, many did not believe that treatment helped their AKs, and inability to assess effectiveness due to premature discontinuation secondary to AE was a common theme. Most stated that they would continue to seek alternative treatment for AKs in the future. Nonadherence is a major cause of treatment-resistant disease. Shared patient-physician decision-making focusing on the benefits and risks of treatment, realistic expectations, therapeutic alternatives, and counseling may increase adherence and improve outcomes.

## Abbreviations

5-FU: topical 5-fluorouracil
AE: adverse event
AK: actinic keratosis
HCP: health care provider
LSR: local skin reaction
